# Subcortical ischemic vascular disease and cognition: A systematic review

**DOI:** 10.1590/S1980-57642009DN20200002

**Published:** 2008

**Authors:** Gilberto Sousa Alves, Carlos Eduardo de Oliveira Alves, Maria Elisa Lanna, Denise Madeira Moreira, Eliasz Engelhardt, Jerson Laks

**Affiliations:** 1 Institute of Psychiatry, Federal University of Rio de Janeiro, Brazil.; 2 Institute of Neurology, Federal University of Rio de Janeiro, Brazil.; 3 Radiology Service of the Procardíaco Hospital, Brazil.; 4 State University of Rio de Janeiro, Brazil.

**Keywords:** neuropsychology, vascular dementia, cerebrovascular disorders, cognition

## Abstract

Subcortical Ischemic Vascular Disease (SIVD) is underdiagnosed. This review investigates the relationship among SIVD severity, cognitive status and neuroimaging markers.

**Methods:**

Cohort, cross-sectional and case control studies were searched on ISI, Medline, Scielo, PsychoInfo and LILACS databases published between 1995 and 2006.

**Results:**

The most impaired cognitive domains were executive, attentional and memory retrieval mechanisms. These cognitive features were frequently associated to White Matter Lesions (WML).

**Conclusions:**

WML is an independent factor in cognitive decline. However, the threshold for this impact is not yet clearly established.

## Abstract (pt)

A doença vascular isquêmica subcortical (DVIS) é uma condição ainda subdiagnosticada. Esta revisão investiga a relação entre a gravidade da DVIS, o status cognitivo e marcadores de neuroimagem.

**Métodos:**

Estudos de coorte, seccionais e casos-controles foram pesquisados nas bases de dados ISI, Medline, Scielo, PsychoInfo e LILACS entre 1995 e 2006.

**Resultados:**

Os domínios cognitivos mais comprometidos foram as funções executivas, a atenção e a recuperação da memória. Estes achados cognitivos estiveram frequentemente associados à presença de lesões em substância branca (LSB).

**Conclusões:**

As LSB constituem um fator de risco independente para o declínio cognitivo. Contudo, o limiar de impacto desta variável ainda não pôde ser claramente estabelecido.

Elderly subjects commonly present with some degree of cerebrovascular lesion on brain Magnetic Resonance Imaging (MRI).^1,2^ Depending on the site, intensity and severity, these lesions may either cause or contribute to further cognitive decline. Among the overall category of cerebrovascular disease (CVD), subcortical ischemic vascular disease (SIVD) is particularly prevalent^3,4^ and encompasses three basic pathological entities: small vessel disease, lacunar infarct and ischemic white matter lesions (WML). This categorization is depicted according to the primary type of brain lesions.^5^

Being a homogeneous construct, the diagnosis of SIVD allows better knowledge of the clinical picture as well as of the natural history, outcome and clinical response to treatment strategies.^5-8^ Regardless of the causal pathological factor of brain vascular lesions, a wide variety of complex mechanisms can be singled out as risk and intervening factors for SIVD. These factors include interactions between vascular etiologies (hypoperfusion caused by arterial stiffness and its relationship to risk factors such as arterial hypertension), microvascular changes in the brain (infarcts, WML, atrophy), host factors (age, education), hypotension, genetic inheritance (CADASIL), and cognitive characteristics.^5,9^

The main clinical manifestations of SIVD may be summarized as a “dysexecutive syndrome” due to the interruption of prefrontal-subcortical loops^10,11^ affecting control, volition, planning, programming, anticipation, inhibition of inappropriate behaviors, and monitoring of complex goal-directed activities.^12,13^ The clinical picture also includes psychomotor slowness, forgetfulness, and changes in speech, affect and mood^11,14^. Despite the fact that dysexecutive syndrome and cognitive decline are frequently observed in SIVD, a debate remains over to what extent the vascular lesions have to be present in order to be responsible for the clinical and neuropsychological picture.^15^ This review aimed to investigate the relationship between SIVD severity and cognitive status with possible neuroimaging markers, addressing two main questions:

(1) how the clinical stage of subcortical disease is related to impairment in specific cognitive domains and how it affects prognosis and functional status;
(2) the impact of the presence, localization and extent of WML on cognition.

## Methods

A systematic review of the literature regarding SIVD was performed by searching data from ISI, Medline, Scielo, PsychoInfo, and LILACS web databases published between 1995 (January) and 2006 (July). The search strategy included key words aimed at investigating a broader spectrum of primary vascular disorders affecting subcortical areas, mainly white-matter lesions: subcortical disease OR dementia, vascular dementia, small vessels disease, Binswanger disease, CADASIL, white-matter disease OR dementia, leukoencephalopathy, cerebrovascular disease or disorders, brain vascular disease OR disorder.

All abstracts were independently read by five authors (GSA; EE; CEOA; MEL; JL) and those studies which complied with the inclusion criteria were selected for further reading. A manual search was also performed to reach articles related to this subject found among the references of the selected studies. The articles which satisfied all the following criteria were included for further reading and analysis. They had (1) to be cohort, cross-sectional, or case control studies with at least one criterion for vascular dementia (DSM-IV, or NINDS-AIREN, or ICD-10, or ADDTC); (2) to provide data on cognitively impaired patients =60 years of age, with or without clinical diagnosis of dementia; (3) to include a comprehensive neuropsychological assessment, as well as at least one neuroimaging exam (brain Computed Tomography or Magnetic Resonance). Studies that described reviews of the literature, case reports, samples with primary psychiatric disorders, exclusively cortical lesions (Alzheimer disease or cortical vascular disease), gray-matter, and cerebellar diseases were excluded from this review. The following items were chosen to describe the correlated variables:

1) methodological characteristics concerning the article;
2) cognitive outcomes and subcortical disease;
3) neuroradiologic findings and cognitive-functional aspects.

To the best of our knowledge, only one other systematic review on the subject has sought to study the relationship between WML and cognition.^16^

## Results

This search retrieved a total of 159 articles, only 32 of which remained eligible according to the inclusion and exclusion criteria. A further analysis excluded three other papers, two because the majority of individuals in the sample were younger than 60 years and one because of absence of neuroimaging to ascertain the presence of SIVD. Tables 1 and 2 show the 29 articles included in this study, displaying two subgroups: firstly those that only studied the cognitive profile (Table 1), plus the studies that assessed the correlations with neuroimaging (Table 2).

**Table 1:** Studies with only clinical and cognitive correlations.

Authors/year	Sample (N)	Design	Cognition and diagnosis of SVI/SVD
Amberla et al.^31^	3 CADASIL groups	CS	Early impairment of EF and later deficits in mental speed and set-shifting abilities
Doody et al.^34^	AD (37) IVD (18)	CS	Lower results on visuoconstructional tasks in IVD group
Frisoni et al.^35^	MCIva (29) MCIad (19) SVD (21)	L	MCI: Poorer performance on frontal tests and more cognitive loss and de­terioration in ADL
Gainotti et al.^38^	AD (68) VaD (40)	CS	Vascular patients had better scores on selective and divided attention and fewer false alarm errors
Galluzzi et al.^36^	MCIam (14) MCIva (29)	CS	Verbal fluency was able to discriminate future conversion to SVD and AD
Mok et al.^28^	SVDinpatients (75) controls (42)	L	Patients with mild cognitive impairment had worse performance on cogni­tive tests than healthy controls
Moretti et al.^20^	SVD (144)	L	Executive function and planning behavior more impaired in SVD patients
Moretti et al.^21^	FLD (40) SVD (40)	L	VaD patients: lower executive functioning, lack of set-shifting, more rigid and apathetic
O'Sullivan et al.^30^	SIVD (32) Controls (17)	CS	Trail Making and Digit Symbol: assess set shifting and were the most dis­criminating tests
Peters et al.^32^	CADASIL (63) controls (40)	L	CADASIL group showed greater impairment in executive functions and processing speed test even in early stages of disease
Pohjasvaara et al.^26^	Post-stroke inpatients (337)	CS	SVD more impaired in executive functions
Tierney et al.^37^	AD (31) SIVD (31)	CS	Verbal recognition memory and verbal fluency distinguished AD from SIVD (with a double dissociation)

AD: Alzheimer's disease; ADL: activities of daily living; CS: cross-sectional; EF: executive functions; FLD: Frontal Lobe Dementia; IVD: ischemia vascular dementia; L: longitudinal; MCI: mild cognitive impairment; MCIad: mild cognitive impairment, Alzheimer; MCIam: mild cognitive impairment, amnestic; MCIva: mild cognitive impairment, vascular; SIVD: subcortical ischaemic vascular disease; SVD: subcortical vascular dementia; VaD: Vascular dementia.

**Table 2:** Studies with correlations among clinical, cognitive and neuroimaging aspects.

Authors/year	Sample (N)	Design	Cognition and diagnosis of SVI/SVD	Neuroimaging and cognition in SVI/SVD
Cohen et al.^33^	VaD(24) Controls (25)	CS	Executive functions and attention strongly associated to the presence and extension of SH	Attention-executive dysfunction cor­related with SH volume but not with whole brain volume (WBV)
de Groot et al.^18^	Population-based sample (1077)	CS	WML more prevalent in women, severity of periventricular WML and subcortical WML correlated with decrease in cogni­tive performance on tests	Association between WML and MMSE
de Groot et al.^19^	Population-based sample (1049)	CS	Worse scores on self report cognitive measures (subjective cognitive failure) in patients with higher WML severity	Correlation between periventricular WML and low scores on subjective cognitive failure questionnaire
Jokinen et al.^25^	Controls (38) Post-strokepatients (238) SIVD (85)	CS	SVD: lower scores in executive functions and delayed recall than OS patients	WML regions were weakly correlated to executive functions
Kramer et al.^27^	controls(27) SVD (12)	CS	SIVD showed lower performance on executive tests - Stroop and California Card Sorting test (conceptual reasoning)	Extent of white matter correlated with executive tests (Stroop interference and fewer sorts in card sorting task). No differences in global cognitive abilities
Libon et al.^23^	AD (16) SVD (14)	CS	IVD: lower learning across trials of PRLT and greater DI on CVLT	Higher leucoaraiosis correlated with decrease on test of psychomotor skills
Libon et al.^22^	AD (33) SVD (27)	CS	SVD: Lower intrusion errors and better recognition and cued recall	WML correlated with decrease on ex­ecutive performance
de Mendonça et al.^41^	MCI (40)	CS	No differences in cognitive tests between patients with subcortical features and those without	Presence of WML not associated with cognitive or specific neuropsychiatric characteristics
Moser et al.^24^	SVD (24) VaD (9)	CS	Association between some executive functions and the presence of SH	Association between SH and atten­tional-psychomotor speed tasks
Paul et al.^3^	Non demented individuals (106)	CS	Increasing levels of SH were associated with poorer cognitive status among older individuals	Relation SH and cognition was found only in a subset of individuals with larger lesions
Price et al.^39^	AD (34) VaD/SVD (35)	CS	No correlation between MMSE and presence of WML	An increase of WMA was associated with greater impairment on measures of executive control and VCA
Prins et al.^15^	Population-based sample (1077)	L	Measures of small vessel disease by MRI associated with cognitive decline in ex­ecutive functions and processing speed	Periventricular WM changes associated to decline in processing information/executive functions
Reed et al.^43^	AD (8), VD (5), MD (7), controls (31)	CS		WML and lacunes correlated with ex­ecutive dysfunction
Sabri et al.^40^	SVD (57)	CS	WML did not correlate with cognitive im­pairment or to neuropsychological tests	No correlations
Skoog et al.^17^	DA (35) VaD (55) controls (134)	CS	WML correlated with general and spe­cific cognitive domains (VCA and pro­cessing speed)	WML correlated with impaired cogni­tive functioning in both non-demented and demented groups
Van Swieten et al.^42^	SVD (44)	CS	No associations between MMSE and CAMDEX scores and the degree of WML	More diffuse WML were associated to poorer performance on executive and VCA tasks
Wen et al.^60^	post-stroke inpatients (257)	CS	Greater WML correlated with age, more lacunar infarcts, more severe pre-stroke cognitive decline	WML but not lacunar infarcts corre­lated with executive tests (in MDRS)

AD: Alzheimer's disease; ADL: activities of daily living; CS: cross-sectional; CVLT: California verbal learning test; DI: discrimination index; IVD: ischaemic vascular dementia; L: longitudinal; EF: executive functions; FLD: Frontal Lobe Dementia; MCI: mild cognitive impairment; MCIva: mild cognitive impairment, vascular; MCIad: mild cognitive impairment, Alzheimer; MCIam: mild cognitive impairment, amnestic; MDRS: Mattis Dementia Rating Scale; MMSE: mini mental state examination; OS: patients with other causes of stroke, non subcortical; PRLT: pursuit rotor learning tests; SIVD: subcortical ischaemic vascular disease; SVD: subcortical vascular dementia; SH: substance hyperintensities; VCA: visuo constructional abilities; VaD: Vascular dementia; WML: white matter lesions .

### Methodological characteristics of the articles

Twenty-four studies were cross-sectional and five longitudinal. The samples assessed in the articles were predominantly outpatients (18), followed by inpatients (6 articles) and mixed samples (inpatients/outpatients) in one article.^17^ Seven articles involved samples from branches of multi-center projects. Three studies consisted of population based individuals,^15,18-19^ whereas four articles were from a study involving outpatients.^20-23^ One article did not describe the source of the patients.^24^

### Cognitive manifestations and subcortical disease

The majority of the articles (n=28, 96.5 %) found a correlation between the clinical diagnosis of SIVD and cognitive alterations. The present analysis focused on executive dysfunction and is complemented by data on other cognitive domains.

### Executive dysfunction 

A worse performance in executive functions was the main finding of the studies comparing SIVD patients with other causes of dementia, such as cortical stroke patients,^25,26^ lacunar infarcts^27^ and AD.^22^

Executive dysfunction was considered a predictor of worse performance in complex activities of daily life in one study.^28^ In the Libon et al.^22^ study, the SIVD group presented greater impairment in the executive control tests, such as the *Boston Revision of the Wechsler Memory Scale-Mental Control Subtest* (WMS-MC),^29^ in relation to AD patients. Impairment in tasks assessing the set shifting process was described in some studies, whether comparing SIVD to healthy controls^30^ or to other groups of patients such as fronto temporal dementia^21^. Worse performance in mental flexibility tasks (Wisconsin Card Sorting Test), together with a lack of response inhibition (Stroop Test) was also seen in a recent article.^25^ In the work by O’Sullivan et al.,^30^ a brief assessment of executive functions involving Trail Making and digit symbols was able to provide good sensitivity and specificity for distinguishing individuals with ischemic leukoaraiosis from subjects with healthy aging.

Impairment of executive functioning at early stages of the disease was investigated in two studies using outpatient samples diagnosed with CADASIL (autosomal dominant arteriopathy associated with subcortical infarcts and leukoencephalopathy). One of these^31^ evaluated three distinct clinical groups – patients with no previous history of cerebral infarct and two groups with recent stroke, with and without vascular dementia (VD). Using MRI, the authors observed impairment in executive functions in the group with mild radiological alterations of white matter. In the other article,^32^ impaired performance in executive tasks, evidenced by a greater proportion of perseverative errors, was shown in the group with vascular cognitive impairment (VCI). Accordingly, these findings should enhance the differential diagnosis between vascular and neurodegenerative diseases, since CADASIL has a clinical presentation with frontal executive impairment at very early stages.

### Cognitive performance other than executive

Attentional deficits were also a frequently reported finding^15,21,24,33-36^ and were strongly correlated to subcortical lesions. Lower scores in attention and concentration tests were seen in vascular groups compared to AD^34^ or healthy controls.^33^ Slowing in processing speed, assessed by the Trail Making Test,^30,32-33^ Block Design^17^ and Letter-Digit substitution test^15^ was more associated to subcortical disease.

Distinct mechanisms of memory impairment differentiated AD from SIVD. SIVD patients tended to show better performance than the AD group on delayed recognition memory with or without the help of clues.^22-23,34,37^ Also, the SIVD group performed better than patients with AD in delayed recall tasks,^34^ as wells as selective and divided attention tasks,^38^ and in relation to the number of intrusive errors, cued recall memory retrieval and visual recognition tests.^22^

Impairment of higher cognitive skills was discussed in addition to executive, memory and attention abnormalities. Visuo-spatial and visuo-construction abilities were assessed by specific tasks, such as the Block Design and Clock Drawing Test and were found to be more impaired in SIVD than in AD^20,34,39^ and healthy controls.^17,31^ One of the studies observed greater impairment of such domains in mild stages of VaD.^34^ Language deficits, described as semantic and phonological abnormalities, were seen in the vascular group in a two-year follow-up study.^20^ The accuracy of verbal fluency (equal to 0.75 under the area of the Receiver Operating characteristic Curve, with a confidence interval of 95%) was considered by another study^36^ as one of the best parameters to discriminate individuals with amnestic complaints from those with mild subcortical disease. Another article observed a double dissociation between AD and the SIVD group, with the latter showing a worse performance in verbal fluency and better scores in verbal recognition.^37^

### Neuroimaging and cognitive aspects

Seventeen studies analyzed the association between WML and cognitive features. Sixteen of these articles (94.1%) found a relationship among neuroimaging, cognition and subcortical disease. The main findings are summarized under the topics below.

### Presence of WML and cognitive performance in healthy individuals

Two studies described an association between WML and the presence of cognitive deficits in samples without a clinical diagnosis of dementia. One of these evaluated oldest old patients (aged ≥85 years) and observed an association between microangiopathy lesions and cognitive decline.^17^ Another article found a worse cognitive performance (measured by ADAS-cog) and a greater intensity of small infarcts in a non-demented group (with Clinical Dementia Rating . CDR 0), in comparison with healthy individuals without radiological alterations.^28^

### The presence of WML and cognitive alterations

There seems to be an association between the attention and executive function tests and the presence of subcortical hyperintensities (SHI) in MRI, although the degree of global cortical atrophy was not related to the occurrence of SHI.^33^ Different studies found similar associations between the presence of subcortical lesions and impaired speed of thought and specific psychomotor skills. A decrease in cognitive processing speed was associated with the presence of WML^17^ and hyperintensities in MRI.^24^ The presence of WML was also associated with lower scores in visuo-constructional,^39^ visuo-motor (Block Design), and visuo-spatial skills (Clock-Drawing Test),^17^ attention and concentration^24,33^ and the severity of the microangiopathic lesions correlated to decreased learning of motor skills.^23^

Interestingly, other cognitive domains were not associated with subcortical lesions. For instance, a greater intensity of WML neither correlated with a more serious impairment of memory and language^39^ nor to global cognitive scores measured by the Mattis Dementia Rating Scale (MDRS).^27^ Two outpatient studies^40-41^ observed no correlation between the severity of WML and performance in neuropsychological tests.

### Localization and extent of WMLs and cognitive alterations

Six studies^15,18-19,23,42-43^ directly analyzed the correlation between the presence and extension of WML and global cognitive performance and specific functions, with different findings.

### Periventricular white matter lesions and cognition

A positive association between the degree of cognitive decline and the presence of periventricular WML (PWML) was observed in four studies.^15,18-19,23^ The presence of PWML, in comparison with diffuse subcortical lesions, was more associated with global cognitive impairment in two studies.^18,19^ A greater severity of PWML was associated with more subjective complaints in the *Cognitive Failure Questionnaire*, a semi-structured instrument which evaluated the patient’s perception of their own cognition.^19^ PWML was associated with specific cognitive skills, executive functions among them,^15,42-43^ and the processing of information.^15^ In one study the association with executive dysfunction occurred independently to the presence of lacunar infarcts.^42^ When compared with microangiopathy in frontal or occipito-parietal areas, diffuse subcortical lesions were also associated with a worse performance in executive tests (Digit Symbol, Trail-Making B).^42^

### Extent of WMLs and cognition

Only one article suggested a threshold of WML related to executive dysfunction. The authors observed that when 50% of white matter was compromised executive dysfunction ensued.^39^

## Discussion

The present review retrieved 29 studies on the issue, most with a cross sectional design (n=24). The limited number of prospective studies assessing the correlations of neuroimaging and cognitive features of SIVD may be explained partly by the high cost and the difficulties inherent to the recruitment and assessment of samples. Furthermore, prospective studies of this disease are often faced with high rates of mortality-morbidity which impact the control of possible biases (attrition, survival or performance).^16^

The majority of the studies (n=15) found a positive clinical association between executive dysfunction and subcortical dementia. Loss of frontal executive control is a major component of cognitive disability^11^ and may also show quantitative and qualitative differences between SIVD and other clinical groups (AD, cortical stroke patients), contributing as a method to validate the diagnostic criteria of subcortical disease, in addition to the assessment by brain imaging currently adopted.

The characteristic pattern of memory impairment presented by SIVD in the selected articles dovetails with previous literature and strengthens the hypothesis of possible different neurological pathways being involved in vascular and Alzheimer’s disease. While retrieval and evocation processes are highly impaired in SIVD because of disruption in executive circuits,^44-46^ recognition with the help of clues (cued recall) is more preserved. On the other hand, encoding is severely impaired and cued recall tasks show a larger number of intrusions in AD.

In three studies,^22,37,38^ the cognitive assessment of Alzheimer and vascular patients demonstrated a pattern of double dissociation and reinforced previous literature outlining the frontal dysfunction as the most characteristic pattern of SIVD.^47,48^ The vascular group showed worse performance in executive control and graphomotor tasks,^22^ whereas they performed better than the AD patients in the assessment of delayed recall tasks with or without the help of clues. Furthermore, a clear dissociation was observed in the early stages of VaD and in AD. The former group showed more impairment of sensory-motor and attentional components whereas the latter presented worse performance on selective and divided attention.^38^

Studies on CADASIL, although few in number, offer an important diagnostic and prognostic model for the characterization of cognitive manifestations in subcortical vascular disease. They can also provide correlations with functional impairment and degree of cerebral lesion. Despite being rare, CADASIL merits interest as it can be considered a pure form of subcortical ischemic dementia. For this reason, longitudinal and multicentric studies could clarify the role of lacunes and WML in mood disorders and psychomotor and cognitive abnormalities in patients with this disease.^49^

Speed of psychomotor processing and some higher cortical functions such as language, visuo-spatial, and visuo-constructional abilities were also frequently reported.^24,33-36^ It is possible that slowing of mental speed may account for lower scores on cognitive tests as an independent variable. Studies with timed and non-timed executive tests have shown that slow performance places greater demands on the retention of information in working memory and associated neural circuits.^30^ As most neuropsychological protocols give emphasis to executive function,^50^ other aspects of neuropsychological functioning may have been underestimated and how language, visuo-spatial and visuo-motor skills can contribute in differentiating SIVD from other groups remains an open issue.

The importance of white matter disease as an isolated factor of cognitive impairment is frequently discussed,^51^ and some observations seem to suggest an effect which might be independent of large infarcts and degenerative lesions of AD.^52^ It is known that WML, like lacunar infarcts, can interrupt subcortical pre-frontal loops, leading to impaired functioning of the frontal lobe and to deficiency in the processing of information.^11,46,53^ There is evidence that periventricular white-matter lesions and subcortical white-matter lesions affect cognitive functions in different ways^54,55^ where some articles have explored this hypothesis. The main difference between these subcortical lesions is based on impaired neural pathways. While the periventricular WML damages long association fibers, in turn connected to distant cortical areas, lesions in short fibers result in the formation of more diffuse WML. Thus, WML abnormalities could compose disconnection syndromes, with diffuse WML more related to the interruption of local networks, whereas the periventricular lesions would lead to the impairment of functions which require coordination of multiple cortical areas distant from one another.^12,56^

One study found a threshold of WML for executive dysfunction,^39^ but the other twenty six drew no conclusion on this issue. According to the author, important limitations were associated to this finding. Although neuroimaging has a leading role, any proposition of a more measurable criterion for subcortical dementia has to balance not only the percentage of white matter lesions measured but also clinical and cognitive features, such as the influence of comorbid diseases (e.g., mixed dementia) and measures of other cognitive functions (memory and language impairments).^39^

In the majority of articles which included neuroimaging assessment (88.2% or 15 in 17 papers), the presence of subcortical lesions was strongly correlated to executive dysfunction and also with impairment in attention and visuo-constructional abilities and psychomotor slowing (see Tables). However, evidence regarding the role of extension and localization of WML in cognitive and functional dysfunctions showed conflicting results. This limitation is probably due to methodological issues and to the difficulties in differentiating the cognitive effects of concurrent pathologies, such as cortical lesions in AD. The former is related to differences in the settings of the studies and in socio-demographic variables, the small size effect on cognition of mild WML,^57^ the limited number of longitudinal studies and the short period of follow-up,^58^ and finally, differences in the criteria defining risk factors for cerebrovascular disease^59^ have hindered sound conclusions. In addition, inconsistent results have been collected because of the vast difference in methods of quantifying WML. Technical questions related to the acquisition and quality of cerebral images obtained by CT or MR, as well as the variations in the image contrast, resolutions and angles should also be noted.^60^ In relation to the latter aspect, post mortem studies have evidenced that typical AD pathology was associated to executive deficits in mild stages of dementia and coexisted with small vessel disease.^61^ Thus, it is possible that in some cases the impact of regional white matter lesions on cognition could be overestimated and not consider a developing degenerative process.

Concluding, the concept of subcortical dementia, however well-characterized in terms of neuropsychological alterations, still awaits definition of concomitant factors and overlapping conditions, such as the presence of white matter lesions, small artery lesions (not always easily identifiable by neuroimaging), cortical atrophy and degenerative lesions. In spite of this, the majority of the studies pointed to white matter lesions as an independent factor in cognitive decline. There appears to be a threshold for the impact of this effect and although this has not yet been clearly established, it seems certain that specific frontostriatal circuits are impaired in the process.
